# Orientin: a comprehensive review of a promising bioactive flavonoid

**DOI:** 10.1007/s10787-025-01690-5

**Published:** 2025-03-08

**Authors:** Mohamed I. Fahmy, Mohamed A. Sadek, Kareem Abdou, Ahmed M. El-Dessouki, Riham A. El-Shiekh, Samar S. Khalaf

**Affiliations:** 1 Department of Pharmacology and Toxicology, College of Pharmaceutical Sciences and Drug Manufacturing, Misr University for Science and Technology (MUST), Giza, Egypt; 2 Department of Pharmacology and Toxicology, Faculty of Pharmacy, Cairo University, Cairo, 11562 Egypt; 3 Department of Pharmacology and Toxicology, College of Medicine, University of Arkansas for Medical Sciences, Little Rock, AR 72205 USA; 4 College of Pharmacy, Al-Ain University, Abu Dhabi, United Arab Emirates; 5 Biochemistry Department, Faculty of Pharmacy, Cairo University, Cairo, Egypt; 6 Pharmacology and Toxicology Department, Faculty of Pharmacy, Ahram Canadian University, 6th of October City, Giza, 12566 Egypt; 7 Department of Pharmacognosy, Faculty of Pharmacy, Cairo University, Kasr El-Aini Street, Cairo, 11562 Egypt; 8 Biochemistry Department Faculty of Pharmacy, Heliopolis University, Cairo, Egypt

**Keywords:** Orientin, Flavonoid C-glycoside, Pharmacological activities

## Abstract

Medicinal herbs continue to play an important part in modern drugs and healthcare because customers think that most of them have fewer or milder side effects than traditional modern medicines. Bioactive compounds are typically isolated from plants before being used as a source of therapeutic medicines. As a result, extracting bioactive compounds from medicinal plants is an important step in developing plant-based medications. Orientin is a flavonoid C-glycoside found in many plants, is frequently used in bioactivity studies due to its numerous beneficial properties, which include antioxidants, antiaging, anti-inflammation, vasodilation and cardioprotective, neuroprotective, antidiabetic, hepatoprotective, and adaptogenic effects. In this review, the comprehensive search for the health benefits of orientin was traced. The findings reflected that orientin could be considered one of the important natural candidates as a potential nutraceutical. This underscores its promising attributes and potential applications in health and wellness. Further research may be guaranteed to fully elucidate its benefits and mechanisms of action.

## Introduction

**Fig. 1:**
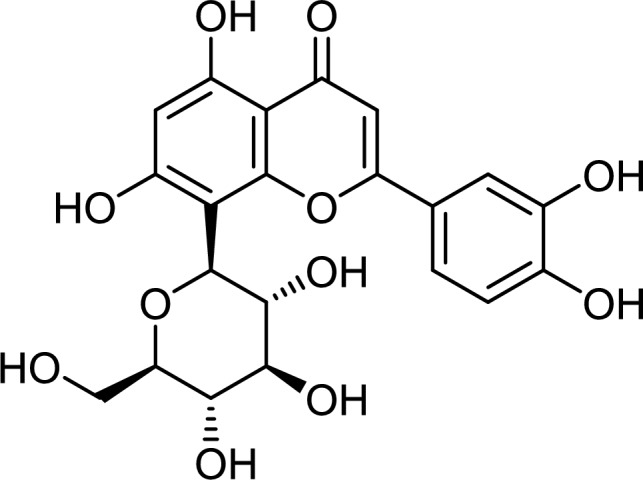
Chemical structure of orientin

Appropriate levels of inflammation play an important role in sustaining health, but excessive inflammation can cause insulin resistance, obesity, and other issues (Cox et al. 2015). Natural anti-inflammatory compounds derived from medicinal plants have been widely utilized to prevent and treat a variety of ailments, with less side effects and increased therapeutic advantages. Dietary flavonoids, particularly glycosides, are the most important phytochemicals in diets and are of great interest due to their wide bioactivity (Yu et al. 2022). In plants, almost all natural flavonoids occur as either *O*-glycosides or *C*-glycosides. The dietary flavonoid C-glycosides have received less attention than their *O*-glycoside counterparts (Xiao et al. 2016). Orientin is a water-soluble flavonoid *C*-glycoside with the IUPAC designation 2-(3,4-dihydroxyphenyl)-5,7-dihydroxy-8-[(2*S*,3*R*,4*R*,5*S*,6*R*)-3,4,5-trihydroxy-6-(hydroxymethyl)oxan-2-yl]chromen-4-one. The chemical formula is C_21_H_20_O_11_, with a molecular weight of 448.3769 g/mol (Koeppen and Roux 1965). The chemical composition of orientin (Fig. 1) reveals that it is largely composed of phenol groups, with two ether groups and one ketone group. To extract orientin from medicinal plants, a polar solvent such methanol, ethanol, or water is necessary (Lam et al. 2016). Orientin has been isolated from a variety of medicinal plants, including *Ocimum sanctum*, *Phyllostachys* species (bamboo leaves), *Passiflora* species (passion flowers), *Trollius* species (Golden Queen), and *Jatropha gossypifolia* (Bellyache Bush) (Lam et al. 2016). Orientin, a possible anti-inflammatory drug, has been shown to ameliorate lung injury by reducing Lipopolysaccharide (LPS)-induced inflammatory responses (Xiao et al. 2021). In osteoarthritic mice, orientin reduced the inflammatory response to ameliorate the condition (Xia et al. 2023). Orientin has been demonstrated to have a variety of bioactivities such as anti-inflammatory, anti-glycation, neuroprotective, antioxidant, antibacterial and radioprotection (Vrinda and Devi 2001; Lee et al. 2006; Bouchouka et al. 2012; Yu et al. 2015b; Yang et al. 2024). The beneficial effects of orientin are discussed in detail in this review.

### Orientin and bone disorders

Bone disorders are diverse groups of disorders that affect bone tissue, cartilage and bone metabolism. Osteoporosis and osteoarthritis are among bone disorders that involve defect in osteoblast and osteoclast formation and activity (Vrathasha et al. 2019). Osteoclasts causes bone resorption by disrupting bone tissue. Osteoclastogenesis requires macrophage colony-stimulating factor (M-CSF) and receptor activator of nuclear factor kappa-Β ligand (RANKL) which are necessary for osteoclast differentiation, development and survival (Herbert et al. 2015; Yu et al. 2015a). Orientin sustains bone health through preventing osteoclast-mediated resorption and promoting osteoblast-mediated formation (Zheng et al. 2023a). Recently, several studies have shown orientin to mitigate bone disorders through suppressing osteoclastogenesis (Nayak and Devi 2005; Gou et al. 2018; Zheng et al. 2023a). Previous study showed that orientin downregulated RANKL signaling, hence inhibiting the differentiation and activity of osteoclasts which in turn maintain bone density and strength. By suppressing RANKL binding to its receptor RANK, orientin inhibits the activation of nuclear factors of activated T-cells 1 (NFATc1), the main regulator of osteoclast differentiation (Yu et al. 2015a). Furthermore, it has been shown that orientin prevents inflammation in chondrocytes in rat model of osteoarthritis, therefore, it attenuates cartilage degradation. This protective effect is mediated through the modulation of the nuclear factor erythroid 2–related factor 2 (Nrf2) and nuclear factor kappa-Β (NF-κB) (Xia et al. 2023). Orientin inhibited NF-κB signaling while stimulated the expression of Nrf2, leading to reduced expression of inflammatory mediators (Zhang et al. 2020; Zheng et al. 2023a).

In addition to its anti-resorptive action, orientin exerts anabolic effects via enhancing bone formation by increasing osteoblast differentiation and activity. Mechanistically, orientin induces the Wnt/β-catenin signaling pathway which promotes the osteoblast mineralization in human, hence enhancing bone formation and remodeling (Nash et al. 2015).

Finally, emerging studies suggest that orientin improves bone mineral density (BMD) and mechanical strength through estrogen receptor pathways, indicating its therapeutic potential for postmenopausal osteoporosis (Nash et al. 2015; Kuriya et al. 2019).

### Orientin in inflammatory and oxidative stress disorders

Orientin exerts its anti-inflammatory action by decreasing the secretion of pro-inflammatory cytokines. It diminished the immunoglobulin E (IgE)-mediated mast cell degranulation by decreasing intracellular calcium level (Dhakal et al. 2020). When orientin was given orally, it suppressed the IgE-mediated passive cutaneous anaphylaxis (PCA) reactions (Dhakal et al. 2020). Previous studies showed that orientin downregulates the NF-κB pathway, which is essential for the transcription of pro-inflammatory cytokines. Orientin prevents NF-κB translocation to the nucleus, limiting cytokine synthesis like TNF-α, IL-6, and IL-1β by inhibiting IκB kinase (Wang et al. 2017; Xiao et al. 2017; Vasudevan Sajini et al. 2024). This mechanism has been confirmed using RAW 264.7 macrophages (Li et al. 2020a) and rodent arthritis models, where orientin effectively suppresses oedema and inflammatory infiltration (Yu et al. 2015a; Gou et al. 2018; Ji and Xu 2022).

Several studies proved that orientin reduces the expression of inducible nitric oxide synthase (iNOS) and cyclooxygenase-2 (COX-2) (Xiao et al. 2017; Xiao et al. 2021), hence, reducing the production of pro-inflammatory mediators such as nitric oxide (NO) and prostaglandins (Zhou et al. 2014; Li et al. 2022b; Song and Fan 2024).

In addition, orientin influences the nuclear factor-kappa B (NF-κB) pathway (Wang et al. 2017; Xiao et al. 2017; Zhang et al. 2020), a central regulator of inflammation. By inhibiting NF-κB activation, orientin decreases the transcription of various pro-inflammatory cytokines, including tumor necrosis factor-alpha (TNF-α) and interleukins (Wang et al. 2017). This modulation leads to a reduction in inflammatory responses in various cell types, including macrophages and chondrocytes (Li et al. 2020a; Xia et al. 2023).

Mitogen-activated protein kinase (MAPK) is targeted by orientin. It attenuates p38 MAPK and JNK activation which are crucial for inflammation-mediated reactions (Vasudevan Sajini et al. 2024). Orientin has been shown to alleviate psoriasis and rheumatoid arthritis through disrupting MAPK signaling (Ji and Xu 2022; Long et al. 2024). In addition to its anti-inflammatory role, orientin has antioxidant effects that arise from its polyphenolic structure, which facilitates the scavenging of hydroxyl and superoxide radicals (Li et al. 2020a; Xiao et al. 2021). By donating hydrogen atoms, it diminishes lipid peroxidation and DNA damage.

The antioxidant action of orientin is attributed to its capacity to scavenge free radicals and improve endogenous defense mechanisms. Orientin neutralizes reactive oxygen species (ROS), thereby preventing oxidative stress-induced cellular damage (Law et al. 2014a; Qi et al. 2018). Furthermore, orientin upregulates the expression of antioxidant enzymes such as superoxide dismutase (SOD) and glutathione peroxidase (GPX) (Xiao et al. 2018, 2021, 2022). Consequently, orientin can mitigate oxidative stress, contributing to its protective effects against inflammation and oxidative damage.

Moreover, orientin regulates endogenous antioxidant pathways, significantly activating Nrf2 transcriptional activity (Li et al. 2022b). This activation facilitates the upregulation of genes encoding antioxidant enzymes such as heme oxygenase-1 (HO-1), catalase, and superoxide dismutase (Xiao et al. 2018), thereby promoting cellular resilience against oxidative stress. The radical scavenging action of C-glycosides when eaten through diet needs to be investigated, as this could widen their therapeutic potential. The in vitro radical scavenging activity of these flavonoid C-glycosides is mainly dependent on structural determinants such as molecular size, degree of glycosylation, hydroxylation pattern, presence of: double bond between C-2 and C-3 that is conjugated to the 4-oxo group, C-4 carbonyl group, flavonol nucleus, the C-3 hydroxyl group, the degree of conjugation with other polyphenols, and their interactions with surrounding molecules (Shen et al. 2007; Amić and Lučić, 2010; Lespade and Bercion 2012).

### Orientin and aging

Orientin has shown a potential as anti-aging agent via its antioxidant and anti-inflammatory effects (An et al. 2012; Jing et al. 2020). Through scavenging ROS and ameliorating oxidative stress, orientin protects cellular components, including DNA, proteins, and lipids, from damage that accelerates aging. In Caenorhabditis elegans, it has been demonstrated that orientin prolongs lifespan, improves locomotion and enhance stress resistance, suggesting its potential to modulate aging processes (Qu et al. 2022).

In addition, orientin influences signaling pathways associated with aging. It has been observed to activate the Nrf2 pathway, leading to the enhancement of antioxidant response and cellular defense mechanisms (Xiao et al. 2018; Li et al. 2022b; Vasudevan Sajini et al. 2024). Moreover, orientin’s interaction with SIRT6 suggests a role in modulating genomic stability and metabolic regulation, both critical factors in aging (Mthembu et al. 2021). In parallel, orientin also stimulates AMP-activated protein kinase (AMPK), facilitating mitochondrial biogenesis and autophagy processes essential for cellular rejuvenation (Liu et al. 2016a; Xiao et al. 2022; Zhang et al. 2022; Elekofehinti et al. 2023; Liu et al. 2024b).

In mammalian models, orientin interacts with sirtuins, particularly SIRT1 and SIRT6. Through upregulating SIRT1, orientin enhances FOXO-mediated transcription, a key factor in oxidative repair and cell survival (Qu et al. 2022; Zhang et al. 2022). SIRT6 activation reduces DNA damage by stabilizing telomeres and promotes chromatin remodeling to regulate metabolic homeostasis. These findings underlie orientin's potential for opposing age-associated metabolic disorders.

In addition, orientin may decrease wrinkles and skin thinning caused by UV exposure through suppressing matrix metalloproteinase (MMP) production (Narayanaswamy et al. 2016; Taherkhani et al. 2020; Ji and Xu 2022), protecting collagen integrity (Long et al. 2024). The inhibition of advanced glycation end products (AGEs) further solidifies its role in mitigating skin aging (Fernandes et al. 2019).

### Orientin and cardiovascular disorders

Orientin, a flavonoid monomer that is present in a variety of medicinal plants, is frequently employed in clinical settings to prevent and treat cardiovascular diseases (CVD). It exhibits a broad spectrum of anti-oxidative, antiarrhythmic, anti-apoptotic, and antithrombotic properties (Thangaraj et al. 2019; Tian et al. 2019; Ong et al. 2022).

#### Vasodilation and cardioprotective effect of orientin

At present, hypertension is one of the most prevalent CVD, which elevates the likelihood of congestive heart failure. However, the maximum efficacy of antihypertensive medications is only 60%, which requires the combination of two or more agents from the classes of angiotensin-converting enzyme or receptor inhibitors, calcium channel blockers, adrenergic agents, diuretics, and vasodilators to achieve optimal effectiveness in patients. As a result, vasorelaxants are essential medications for hypertensive individuals to reduce their elevated blood pressure and protect them from cardiovascular disease (Lam et al. 2016).

Orientin has been shown to induce vasodilation in excised thoracic aortic rings from the New Zealand rabbit. Orientin had an IC50 value of 2.28 μM and 7.27 μM in inducing relaxation of phenylephrine-induced contractions in endothelium-intact and endothelium-isolated aortic rings, respectively. Orientin may function as a vasorelaxant on thoracic aortic rings via the nitric oxide-cGMP pathway, whereas in vascular smooth muscle, it induces relaxation by activating voltage-dependent calcium channels (Fu et al. 2005; Lam et al. 2016; Loh et al. 2020).

Arrhythmia is a common cardiac disorder characterized by an abnormal rhythm of the heartbeat and originates from disturbances in the functioning of the heart and electrical conduction. Arrhythmias have complicated mechanisms and are connected to other CVD that result in heart failure and sudden death (Saber and Abotaleb 2022).

A prospective anti-arrhythmia drug, orientin has the potential to dilate cardiac arteries by inhibiting LTCCs (Trettel et al. 2021). For instance, orientin (3 mg/kg) has the capability to extend the occurrence of ischemia reperfusion (I/R) injury-induced arrhythmia from 0.9 min to 7.6 min, decrease the duration of arrhythmia from 18.2 min to 4.5 min, and lower the rates of mortality, ventricular tachycardia, and ventricular fibrillation from 60%, 90%, and 80% to 10%, 20%, and 10% respectively. Furthermore, orientin at concentrations of 3 μM, 10 μM, and 30 μM can alter the INa-P from − 81.5 pA/pF to − 74.4 pA/pF, − 63.1 pA/pF, and − 55.3 pA/pF, respectively. Orientin (3 mg/kg) has the capability to reduce the QT interval from 175.4 ms to 95.4 ms. In addition, it can extend the occurrence times of VPB, ventricular tachycardia, and ventricular fibrillation from 19.6 s, 38.6 s, and 87.2 s to 45.3 s, 58.5 s, and 170.3 s, respectively (Xiao et al. 2023).

Furthermore, researchers discovered that orientin could alter the kinetic characteristics of cardiomyocytes’ channels and inhibit ionic currents (INa, ITo, and ICaL) (Li et al. 2017a). Orientin is a promising antiarrhythmic drug (Wang et al. 2021; Kimura et al. 2022). However, there are few reports on its anti-arrhythmic effects.

A series of molecular and cellular remodeling, such as inflammatory response, cardiac hypertrophy, and fibrosis, is induced by cardiomyocyte necrosis and apoptosis (Bhatt et al. 2017). In the first instance, these cardiac alterations have a compensating effect, preserving normal cardiac function (Schirone et al. 2017). Conversely, prolonged stress induces cardiac remodeling, resulting in gradual and irreversible heart malfunction, ultimately leading to chronic heart failure and mortality (Prabhu and Frangogiannis 2016).

Oxidative stress is a key factor in the progression of heart disease to heart failure throughout the cardiac remodeling process (Jain et al. 2015). Several cytotoxicities, including DNA damage, lipid peroxidation and protein oxidation, are induced by excessive oxidative stress. These cytotoxicities result in changes in calcium-transport proteins and the activation of hypertrophy signaling pathways, which in turn trigger fibroblast proliferation, cardiomyocyte dysfunction and apoptosis (Azevedo et al. 2015; Liu et al. 2016b). Consequently, it appears that targeting oxidative stress may be a viable approach to the treatment of cardiac remodeling.

Myocardial ischemia (MI) has a complicated etiology that includes several potential causes of ischemic heart disease. Intracellular calcium excess, inflammatory reactions, oxidative stress, apoptosis, and energy metabolism abnormalities are the main contributors to these variables (He et al. 2022). Oxidative stress mainly derives from overproduction of oxygen-free radicals and makes a significant contribution to the development of myocardial ischemia diseases (Xiang et al. 2021).

Orientin was shown to safeguard against myocardial ischemia–reperfusion injury (MIRI) and cardiomyocyte hypoxia/reoxygenation damage via modulating autophagy (Liu et al. 2016b). As is well known, autophagy is a key catabolic mechanism in mammalian cells that breaks down and recycles organelles and macromolecules. Because the heart is made up of terminally differentiated cardiomyocytes, autophagy normally operates at the basic level and helps maintain intracellular homeostasis by eliminating damaged organelles and long-lived or excess protein aggregates. Autophagy is directly linked to several CVD, including MIRI, under certain clinical circumstances (Mei et al. 2015).

In the ischemia–reperfusion (IR) model of the heart, orientin demonstrated the capacity to safeguard cardiomyocytes from ischemic damage and to block their death. It exhibited anti-apoptotic effect in IR damage by neutralizing superoxide anions and hydroxyl radicals in vitro (Xiang et al. 2021).

Orientin attenuated MIRI-induced cardiac hypertrophy, fibrosis, and inflammation, thus inhibiting cardiac remodeling after MI. Moreover, orientin ameliorated oxidative stress through increased eNOS activation and nitric oxide production, which might be some of the mechanisms underlying its cardioprotective effects (Li et al. 2017a).

In addition, orientin treatment raised AMPK and Akt activation, decreased mTOR phosphorylation and Raptor expression, and improved Beclin 1–Bcl-2 interaction in endoplasmic reticulum via increased Beclin 1 phosphorylation and decreased Bcl-2 phosphorylation. Therefore, orientin's cardioprotective benefits during MIRI could be mediated via modulating signaling pathways related with Bcl-2, AMPK, Akt, and mTOR, which in turn regulate autophagy (Liu et al. 2016b).

SIRT3, a NAD + -dependent deacetylase, plays a critical role in MIRI by deacetylating a variety of proteins, such as SOD2 and FOXO3 (Chang et al. 2019). SOD2, which is regulated by reversible lysine acetylation mediated by SIRT3, occupies a key position in this network, indicating that lateral aortic constriction activates SIRT3/SOD2-dependent pathways, which may reduce myocardial autophagic cell death (Ma et al. 2021).

Increased SOD2 acetylation by SIRT3 depletion results in severe oxidative stress, hypertension, and endothelial dysfunction (Dikalova et al. 2017). Recent research suggests that the SIRT3/SOD2 pathway can be modulated to mitigate oxidative stress and apoptosis in MIR, protecting myocardial cells (Zhai et al. 2017). This demonstrates how SIRT3 controls SOD2, which is crucial in the treatment of ischemic heart disease.

Orientin enhances H_2_O_2_-induced SIRT3 and SOD2 reduction, lowers the high AC-SOD2/SOD2 ratio, and 3-TYP counteracts this action. So, it has therapeutic potential for CVD by protecting myocardial cells via the SIRT3/SOD2 pathway (Liu et al. 2024a). Table 1 shows a summary of recent papers about cardioprotective effect of orientin.

**Table 1:** Summary of recent papers for cardioprotective effect of orientin

References	Experimental model & design	Cardiovascular focus	Mechanisms explored
Li et al. (2022a)	In vivo: Mice treated with d-GalN/LPS-induced liver injury In vitro: HepG2 cells exposed to oxidative stress	Indirectly Related (Oxidative stress mechanisms relevant to cardiovascular health)	Nrf2/Keap1 pathway activation; downregulation of oxidative damage markers (MDA, MPO)
Li et al. (2020b)	In vivo: Tissue pharmacokinetics in normal vs. MI rats treated with Polygonum orientale extract	Myocardial ischemia	Tissue distribution of orientin and metabolites
Kong et al. (2020)	In vitro: High glucose (HG)-induced podocyte apoptosis model	Chronic cardiovascular conditions (e.g., diabetic nephropathy, diabetic cardiomyopathy)	Mitophagy regulation; mitochondrial integrity
Xiao et al. (2018)	In vitro: H2O2-induced oxidative damage models	General oxidative stress (relates to cardiovascular injury)	Nrf2/HO-1 signaling activation; MAPK/Akt pathways
Gao et al. (2024)	In vitro: Human endothelial cells treated with ox-LDL	Atherosclerosis	SESN1-mediated autophagy; NF-κB signaling inhibition
Li et al. (2020b)	In vitro: RAW 264.7 macrophages treated with ox-LDL	Atherosclerosis	NF-κB; CD36 downregulation; ROS inhibition; eNOS activity
Ma and Han (2018)	In vivo: MI in mice via LAD ligation; 14-day orientin treatment	Myocardial ischemia	PI3K/Akt and MAPK pathways; fibrosis and hypertrophy biomarkers
Li et al. (2017b)	In vivo: MI mouse model (LAD ligation), treated with 40 mg/kg Orientin for 25 days. In vitro: Hypoxia-induced neonatal rat cardiomyocytes	Myocardial ischemia	eNOS/NO signaling; anti-inflammatory and anti-apoptotic (Bax/Bcl-2 ratio); oxidative stress reduction

### Orientin and central nervous system (CNS)

**Fig. 2:**
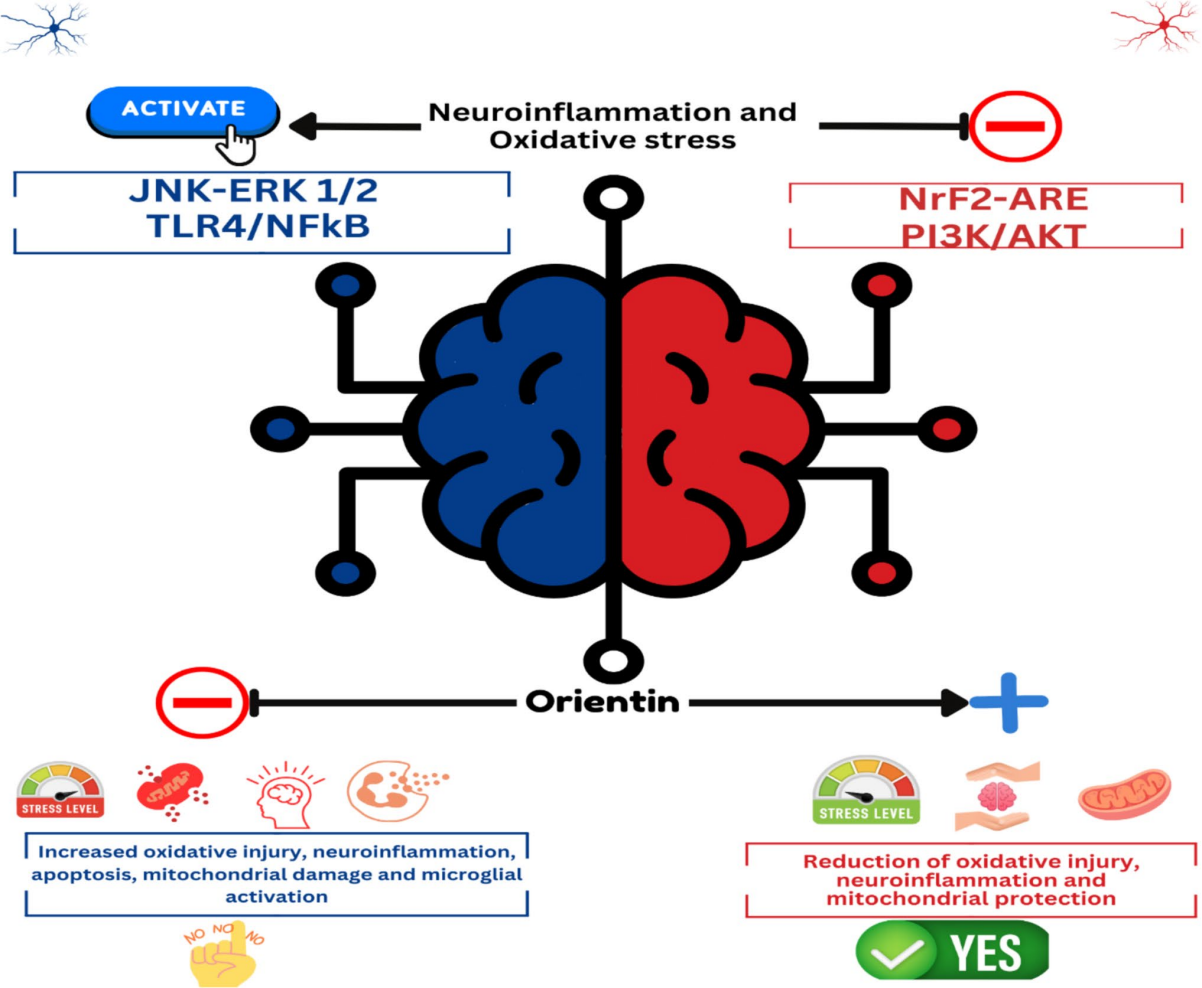
Neuroprotective effect of orientin

For the pharmacological application on central nervous system (CNS), orientin exerted neuroprotective effect on H_2_O_2_-induced apoptosis in SH-SY5Y cell lines with few cytotoxic effect in vitro (Law et al. 2014b). Orientin promoted cognitive recovery and alleviated oxidative stress in Aβ1-42-induced Alzheimer’s disease (AD) rat model (Yu et al. 2015b). It also has been found that orientin accelerated alleviation of depression-like behavior in chronically stressed mice (Liu et al. 2015b), and it improved noise-induced cognitive impairments in a mouse model (Wang et al. 2016). In addition, orientin was reported to regulate TLR4/NF-κB/TNF-α signaling in rats to protect against cerebral ischemia/reperfusion injury (Wang et al. 2017). Figure 2 summarize the neuroprotective role of orientin.

The CNS shows specific vulnerability to injury by free radicals generated as a consequence of oxidative stress compared with peripheral tissues. This predisposition may be attributed to various reasons such as increased consumption of oxygen, high lipid content, and a relative deficiency of antioxidant enzymes. In addition, chronic stress can result in oxidative stress and excessive generation of free radicals which may result in cognitive impairment (Guo et al. 2018).

Oxidative stress significantly contributes to the development of chronic stress-induced depression, in addition to impairing neurotransmission and neuroplasticity. The administration of orientin resulted in a notable improvement in the depression-like behaviors provoked by chronic stress in the murine model, which included symptoms such as anhedonia, diminished locomotor activity, and hypomotility. Orientin treatment was found to normalize the oxidative stress markers and increased the levels of norepinephrine, serotonin and neurotrophin BDNF, and synapse-associated proteins (SYN and PSD-95) in the hippocampus and prefrontal cortex of chronic unpredictable mild stress (CUMS) mice (Liu et al. 2015b).

#### Neuroprotective effects of orientin

Orientin is a promising neuroprotective agent with potential for the study of neuropathic pain (Lam et al. 2016). Neuropathic pain is a form of pain that is induced by a primary lesion or dysfunction of the nervous system. It impacts 30% of the global populace. It can be caused by a variety of factors, such as traumatic injury, diabetes, chemotherapy medications, cancer compression, and viral disease. It results in problems with the human body’s functionality, including activity restrictions or disability (Koncicki et al. 2017).

Neuropathic pain represents an exaggerated neuroimmune response in the spinal cord, which is mediated by a wide variety of mediators. Pro-inflammatory cytokines, including IL-6 and TNF-α, are directly involved in the potentiation of pain perception and the development of central pain sensitization. Overexpression of cytokines can interrupt nerve healing, lead to neuronal death, or worsen brain injury. Orientin demonstrated efficacy in alleviating neuropathic pain in the behavioral assay conducted on spinal nerve ligation (SNL) rats. Simultaneously, orientin decreased the levels of pro-inflammatory cytokines and modulated SOD, GSH, and MDA. It also suppressed the activation of microglia and astrocytes produced by SNL. The neuroprotective action of orientin was facilitated by the suppression of the TLR4/NF-kappa B signaling pathway (Guo et al. 2018).

Huntington’s disease, Parkinson’s disease, AD, amyotrophic lateral sclerosis, and multiple sclerosis are examples of neurodegenerative disorders (NDDs) that are challenging to treat, may reduce life expectancy, and result in impairment (Buendia et al. 2016).

Numerous pathological factors contribute to NDDs, such as autophagy system disorder, inflammatory injury, unfolded protein response (UPR), apoptosis, oxidative stress, and mitochondrial dysfunction. Oxidative stress and its contribution to the pathological processes of NDDs have garnered considerable attention. Therefore, using medications to eliminate oxidative free radicals or activate the antioxidant defence system would be a highly practical way to treat NDDs (Qi et al. 2018).

A multitude of signaling pathways, such as PI3K/AKT, MAPK and NF-κB, are critically involved in the neuronal apoptosis triggered by oxidative stress. The study conducted by (Zhang et al. 2021) demonstrated that orientin suppressed H_2_O_2_-induced apoptosis in PC12 cells by restoring cell viability, reducing the levels of apoptosis, and improving nuclear morphology. Caspase-3 is the primary end-cleaving enzyme involved in the apoptosis of cells; it cleaves and inactivates the DNA repair enzyme PARP, an enzyme important for DNA repair and the initiation of the apoptosis program. Orientin inhibited caspase-3 activation and PARP degradation, while also decreasing H_2_O_2_-induced phosphorylation of Akt, MAPK, and Src signalling proteins, as well as reducing ROS accumulation.

AD is a common kind of dementia, marked by deterioration in memory, cognitive function, and personality. Pathological characteristics include extracellular beta-amyloid (Aβ) plaques and intracellular neurofibrillary tangles (NFTs) comprised of hyperphosphorylated tau protein. Aβ-induced toxicity and oxidative stress are important elements in its development (JunYi et al. 2023).

Aβ binds to Aβ-binding alcohol dehydrogenase (ABAD), establishing a molecular connection between Aβ and mitochondria. This interaction exacerbates Aβ-induced oxidative stress, disrupts energy metabolism, and contributes to mitochondrial dysfunction in AD models (Valaasani et al. 2014). The progression of mitochondrial dysfunction leads to the opening of permeability transition pores (mPTP), which plays a role in triggering an apoptosis cascade that may hasten early learning and memory deficits in models of AD. Consequently, mitigating mitochondrial dysfunction presents a promising therapeutic approach for AD (Shao et al. 2014).

Moreover, erythroid 2-related factor 2 (Nrf2) is essential for inducing the defense mechanisms of oxidative stress. Brains from patients with AD have been reported to display diminished nuclear Nrf2 in the hippocampus. On the other hand, Aβ toxicity is found to be resisted by the neurons overexpressing Nrf2 through upregulated target genes of Nrf2 and reduced oxidative stress. Orientin promoted cognitive recovery and reduced oxidative stress in the Aβ1-42-induced AD rat model by activating the Nrf2/HO-1 signaling pathway, inhibiting apoptosis induced by Aβ1-42, and improving cognitive deficits (Yu et al. 2015b).

Parkinson’s disease (PD) is a progressive NDD that primarily affects motor function caused by the degeneration of dopaminergic neurons in the substantia nigra. This disorder poses a great challenge, affecting millions of people worldwide, and the symptoms include tremors, postural instability, rigidity, and bradykinesia, which significantly lead to decreased quality of life and increased morbidity (Padhan et al. 2024).

Oxidative stress and neuroinflammation are recognized as central pathological features of PD. Recent findings indicate that the PI3K-Akt, Nrf2-ARE, TLR4/NF-kB, and JNK-ERK1/2 pathways are crucial in the development of PD (Fahmy et al. 2024; Vasudevan Sajini et al. 2024).

In the Swiss albino mouse model of PD and SH-SY5Y cell lines, orientin has exhibited modulatory potential on these pathways. Orientin reduced the expression of NRF2, AKT, and TNF-α genes, as well as behavioral parameters, inflammatory markers (IL-6 and IL-8), oxidative stress markers (ROS, SOD, CAT, and GPX) (Vasudevan Sajini et al. 2024).

### Orientin and diabetes

The prevalence of diabetes is increasing in spite of the revolutionary development the drug development and the rise of various management strategies (Kaveeshwar and Cornwall 2014). It is predictable that in 2030, the number of diabetes mellitus patients will rise to reach 366 million (Doudach et al. 2023). Troublesome also is the depraved economic impact of the disease, which was estimated to be 612-billion USD (Mozaffarian et al. 2016). Presently, the existing antidiabetic agents are associated with numerous adverse reactions including weight gain, hypoglycemia, and gastrointestinal disturbances while the invasive surgical interventions, such asbariatric surgery, pancreatic transplant, islet cells transplantation are not affordable, in addition to having marked failure rate and post-surgical complications (Tiwari and Aw 2024). Collectively, these factors contribute to the increasing interest in the examination of natural products that have potential antidiabetic effects (Madubuike et al. 2023). To do so, lots of effort have been paid to understand the nature of type 2 diabetes (T2DM) and different signaling pathways which can targeted to alleviate the signs and symptoms and offer alternative therapeutic strategies. T2DM is characterized by an inadequate production and release of insulin from pancreatic beta-cells accompanied by remarkable insulin resistance in the target tissues. Insulin resistance is usually associated with high levels of inflammatory and oxidative biomarkers and mediators namely, tumor necrosis factor (TNF)-α, plasminogen activator inhibitor-1 (PAI-1), C-reactive protein (CRP), interleukin-6 (IL-6). An additional connection between inflammation, obesity, and T2DM in which obese patients exhibited an elevated level of cytokines that caused hepatic insulin resistance has been found (Granic et al. 2009; Yeo et al. 2010; Mohamed et al. 2024).

Orientin, one of the isolable bioactive compounds of Fenugreek, is often used in various bioactivity studies due to its extensive beneficial properties, such as antioxidant, anti-inflammation, cardioprotective, neuroprotective, antidepressant-like, anti-adipogenesis, and antinociceptive effects (Kong et al. 2020). Moreover, orientin has obvious anti-inflammatory and antioxidtive effects (Lam et al. 2016). Meanwhile, the oxidative stress pathway in the pathogenesis of diabetes and diabetic nephropathy is very important. Moreover, orientin was found to suppress the transcription nuclear factor-kappa B (NF-κB) (Tian et al. 2019) and it also inhibits NF-κB signaling pathways (Thangaraj and Vaiyapuri 2017b).

In the same context, preclinical studies demonstrated that orientin significantly modulates the overexpression of inflammatory inducible enzymes including inducible nitric oxide synthase (iNOS) and COX-2 (Khalil et al. 2022a).

In addition, orientin was also found to possess antiapoptotic effects that results in cytoprotective actions and increasing cell survival. Orientin regulates and suppresses the migration and production of apoptotic markers such as iNOS, BCL-2 and CYP-1A1 (Khalil et al. 2022a). Meanwhile, there is already evidence indicating that dietary flavones, particularly the C-glycosyl flavone isomers, orientin and isoorientin, can improve glucose metabolism in cultured adipocytes (Ku et al. 2014; Ziqubu et al. 2020a; Ziqubu et al. 2020b), Moreover, orientin increased the expression of Irs1, Pi3k, and Glut2, and increased glucose uptake to potentially alleviate insulin resistance (Mazibuko-Mbeje et al. 2021). Nevertheless, consistent with other bioactive compounds found in rooibos, such as aspalathin, orientin displays a strong potential to enhance the mRNA expression levels of Ampk and Cpt1, which are all part of an essential mechanism involved in insulin- independent glucose regulation (Mazibuko-Mbeje et al. 2020). In addition, effective upregulation of Ampk and Cpt1 could suggest that orientin plays a major role in controlling mitochondrial beta-oxidation and energy metabolism. This is consistent with in vitro biological effects observed with using another dietary flavone (isoorientin) in cultured adipocytes, also exposed to high levels of palmitate. These are all vital molecular mechanisms that are linked with the therapeutic actions of well-known drugs such as metformin, which are accredited for targeting the reversal of hepatic insulin resistance to improve metabolic function (Rena et al. 2017). In agreement, the current communication certainly affirms that orientin improves the substrate utilization within the liver subjected to insulin resistance, in part by regulating essential mechanisms of insulin signaling and energy metabolism (Mazibuko-Mbeje et al. 2021). Alternatively, it was clear that treatment with orientin was able to reverse palmitate-induced hepatic insulin resistance in part by improving substrate utilization, for example, it enhanced glucose uptake and effectively modulated the expression of genes coding for glucose transport (Glut2) and insulin signaling (Irs1 and PI3k) (Stanford and Goodyear 2014).

### Orientin and hepatoprotective effects

**Fig. 3:**
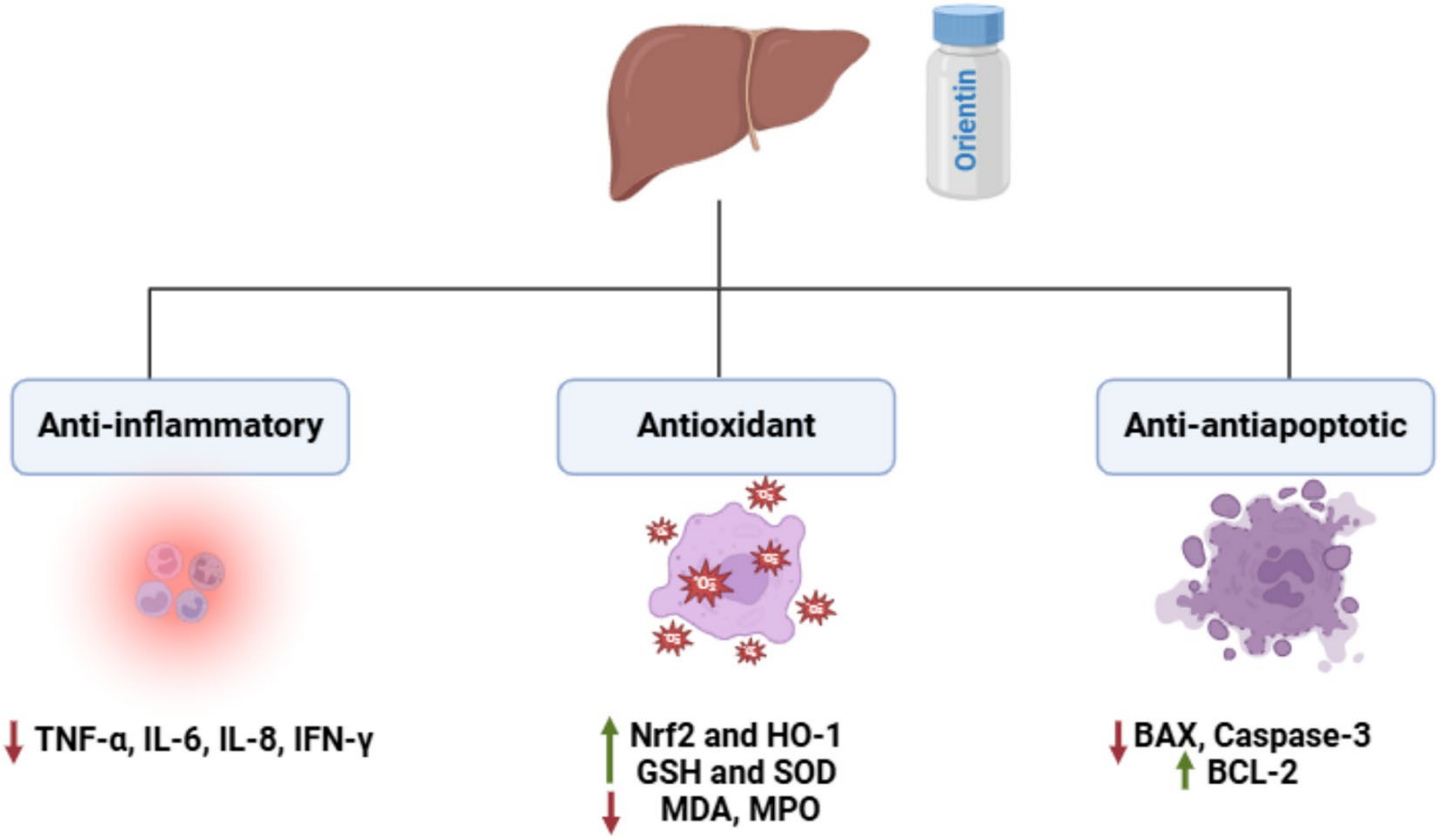
Hepatoprotective effects of orientin

Liver inflammation is one common, widely spread risk factor of liver diseases and is considered the major cause of hepatic tissue damage (Del Campo et al. 2018). Consequently, inflammation leads to various hepatic tissue insults usually characterized by the induction of fibrosis, cirrhosis, primary liver cancer, and death (Calvente et al. 2019; de Haan et al. 2022). Due to its antioxidant and anti-inflammatory activities, orientin has been studied for its hepatoprotective activity. The recent in-vivo study has documented its potential hepatoprotective effects via targeting oxidative biomarkers that increased due to prolonged administration of acetaminophen-induced liver failure model in mice (Xiao et al. 2022). The study reported that orientin could Nrf2 and HO-1 that have free radical scavenging effects and subsequent inhibition of oxidative parameters, including SOD, MPO, and MDA, which further induce liver tissue inflammation and deterioration (Jaeschke et al. 2012). Besides, the study postulated that orientin was found to activate GSH and SOD antioxidant enzymes which results in a marked reduction of liver enzymes AST and ALT indicating prominent alleviation of hepatic tissue inflammation and damage. In addition, the study showed antiapoptotic effects via suppressing the pro-apoptotic proteins, Bax and caspase-3, alongside activating the anti-apoptotic Bcl2, which causes tissue survival and stops cellular apoptosis. Another in-vitro study has investigated the ability of orientin to alleviate liver inflammation in hepatic stellate cells in CCL4 induced liver injury (Xiao et al. 2022). The results obtained from the study demonstrated that orientin primarily downregulated pro-inflammatory biomarkers TNF-α, IL-6, IL-8, and IFN-γ that are markedly increased in CCL4 induced hepatotoxicity model. The hepatoprotective activity of orientin was reflected by decreased ALT and AST liver enzymes (Fig. 3).

### Orientin and cancer

Beyond the previously mentioned pharmacological and therapeutic benefits, researchers highlighted orientin as an extraordinary nutraceutical. Concisely, orientin exhibits a remarkable impact on more complex conditions, including cancer and aging (Lam et al. 2016). In the following subsections, we will underline orientin’s anti-cancer, immunomodulatory, radioprotective, and adaptogenic bioactivity. This section also highlights orientin protective influence against drug-induced toxicity.

#### Anti-cancer and immunomodulatory effects

Orientin regulate the growth of tumor cells and enhance their apoptosis, underscoring orientin as a promising anti-cancer agent (Tian et al. 2019). Besides tumor cell growth, orientin suppress the metastasis of living cells through regulating angiogenesis (Kim et al. 2018). Noteworthy, Orientin showed its anti-cancer effect in numerous types, including bladder carcinoma (Tian et al. 2019), hepatic carcinoma (Sharma et al. 2016), esophageal carcinoma (An et al. 2015), pancreatic carcinoma (Kasperczyk et al. 2009), colorectal cancer (Thangaraj and Vaiyapuri 2017a), and breast cancer (Czemplik et al. 2016; Kim et al. 2018).

The anti-cancer effect of orientin is credited, at least in part, to its ability to induce molecular and cellular alterations. As previously mentioned, orientin is an antioxidant that control glutathione (GSH) homeostasis (Xiao et al. 2018). The latter in turn has a positive influences against cancer. These influences not only because of its antioxidant properties, but also due to its ability to modify cell growth, immunity, and programmed cellular death (Kennedy et al. 2020). Moreover, Thangaraj et al. highlighted the effect of orientin in colorectal cancer. Concisely, orientin acts as an anti-inflammatory agent which suppress inflammatory cytokine levels, alleviating cancer severity (Thangaraj and Vaiyapuri 2017a). In molecular level, orientin regulates extracellular signal-regulated kinase (ERK) activity, as shown in breast cancer cells (Kim et al. 2018). ERK overexpression provokes cellular damage via various pathways, such as organelle dysfunction, DNA destruction, senescence, and metabolic imbalance (Timofeev et al. 2024). Moreover, orientin promotes caspase levels in cancer cells, triggering their apoptosis (Tian et al. 2019).

**Fig. 4:**
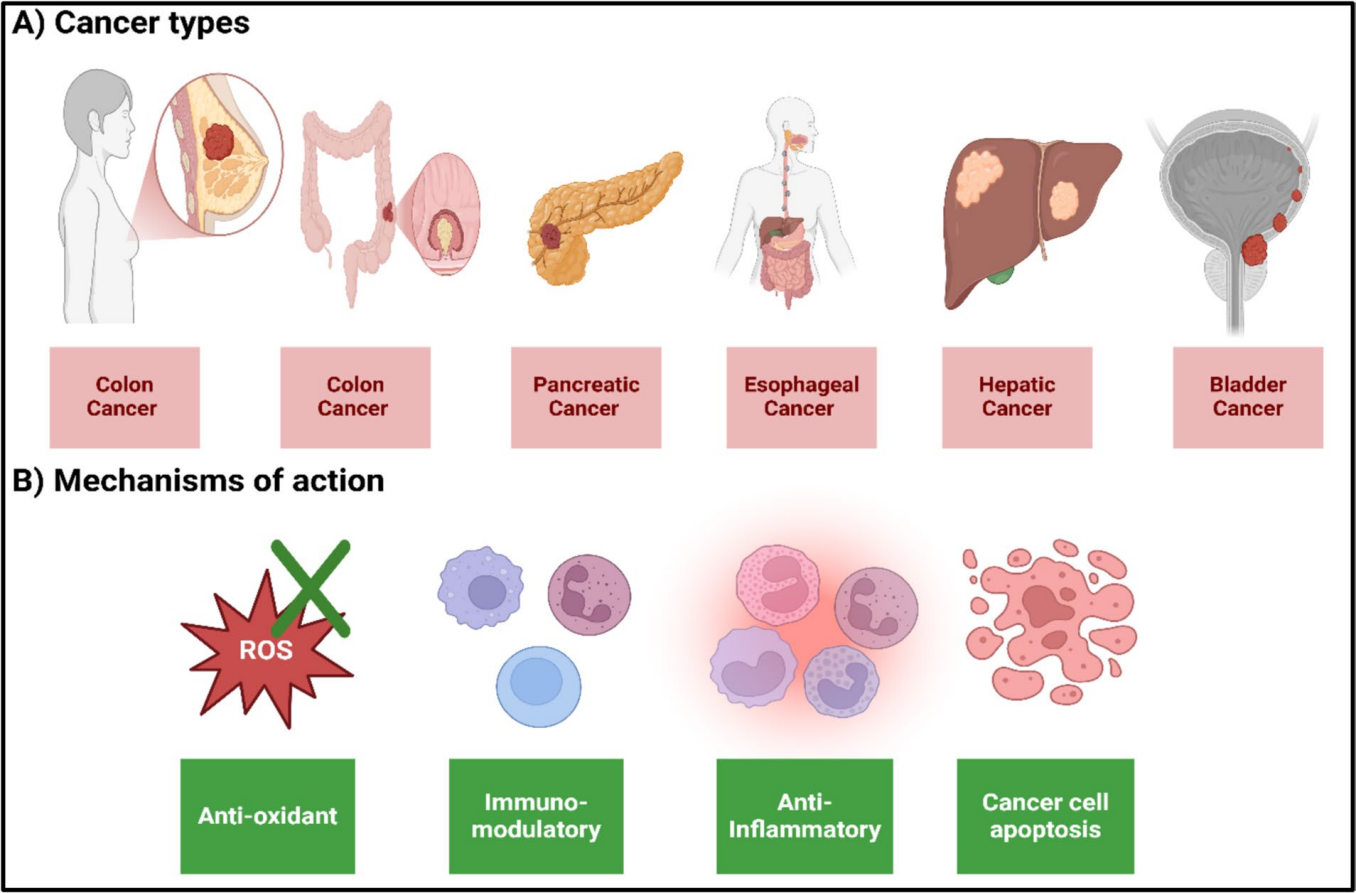
Illustration of various cancer types managed by orientin (**A**) and orientin mechanisms of action in alleviating cancer (**B**)

Besides orientin molecular mechanisms, orientin exerts an immunomodulatory effect. Orientin is documented to establish an anti-inflammatory effect by limiting the recruitment of inflammatory phenotypes of T-cells and macrophages (Long et al. 2024). As previously mentioned, orientin is an anti-inflammatory molecule, and regulating inflammatory cytokines manipulate the activity of T-cells, B-cells, macrophages, and dendritic cells (Al-Qahtani et al. 2024). Figure 4 illustrates the axes that orientin path to establish its anticancer effect in different cancer types.

#### Orientin as a radioprotective agent

Cells exposed to radiation, either medically or through environmental sources, are prone to mutation and apoptosis (Talapko et al. 2024). Subsequently, radiotherapy is used in almost half of patients with localized tumors. However, radiation can negatively harm cells neighboring cancer cells and distant cells through a series of released cytokines (Baskar et al. 2014). Interestingly, Nayak et al. displayed the ability of orientin to protect the bone marrow of mice exposed to radiation, enhancing their survival rate (Nayak and Devi 2005). Inline, orientin-treated mice showed a lower gastrointestinal complication rate following radiation (Uma Devi et al. 1999). Noteworthy, orientin’s radioprotective influence is documented not only in animals but also in human cells. Concisely, orientin protected lymphocytes from gamma radiation-induced chromosomal damage (Vrinda and Uma Devi 2001). Moreover, orientin amended the production of reactive oxygen species in lymphocytes exposed to radiation (Vrinda and Uma Devi 2001). The radioprotective influence of orientin could be justified, at least in part, by its previously mentioned pharmacological effects. Concisely, radiation exacerbates the production of ROS that triggers apoptosis and autophagy of cells (Zheng et al. 2023b); therefore, the antioxidant effect of orientin could minimize cellular death. Moreover, radiation-induced inflammation plays a role in radiation side effects (Schaue et al. 2015); hence, using an anti-inflammatory agent, like orientin, could minimize radiation toxicity. Interestingly, Baliga et al. reported that orientin radioprotection selectively favors healthy cells over cancer cells (Baliga et al. 2016). Based on its safety, efficacy, and selectivity, it points to orientin as a promising radioprotective agent, especially in cancer patients usually exposed to radiation, where it can exert its radioprotective influence and be an anti-cancer.

#### Adaptogenic benefits of orientin

Adaptogens are natural compounds with many effects, including decreasing fatigue and stress, besides improving endurance, muscular strength, memory, and concentration (Amir et al. 2023). Orientin is reported to relieve stress and depression in mice exposed to chronic unpredictable mild stress (CUMS). Orientin improved not only the behavioral symptoms of CUMS, as shown in the sucrose preference test, but also enhanced the levels of serotonin in the prefrontal cortex (Liu et al. 2015a). Moreover, Wang et al. displayed that the oral administration of orientin improves the learning and memory of mice, besides decreasing the level of the stress hormone corticosterone (Wang et al. 2016). In addition, orientin enhances metabolic activity, underscoring its possible utilization as an adaptogen (Mazibuko-Mbeje et al. 2021). Interestingly, a recent study highlighted the beneficial impacts of orientin on skeletal muscles. Briefly, orientin promotes mitochondrial functions and regulates the transition between different types of muscle fibers (Liu et al. 2024b).

#### Orientin mitigates drug-induced toxicity

Unfortunately, the administration of drugs is a double-edged sword weapon, where drugs, on one side, mitigate diseases and, on the other side, trigger side effects. Drug-induced toxicity can provoke morphological and physiological alteration of vital organs, such as the heart, lungs, liver, and kidneys. Furthermore, it can exaggerate oxidative stress and inflammation, which, in turn, damages the cellular component, including DNA (Deavall et al. 2012). Fortunately, the administration of orientin demonstrated a favorable effect in various toxicity models. For instance, the administration of dextran is known to trigger colitis and flare the release of cytokines, which are mitigated by orientin (Sun et al. 2016). Moreover, acetaminophen is one of the most commonly used medications with a well-known hepatotoxicity. Xiao et al. showed that orientin controls the level of the metabolizing enzyme, cytochrome P450 2E1, amending acetaminophen toxicity and mortality rates (Xiao et al. 2022). Also, 40 mg/kg of orientin could augment the levels of antioxidants in the kidney to a level similar to vitamin E (An et al. 2012), pointing to its possible promising effect on nephrotoxicity. In the lungs, orientin suppressed inflammasomes and oxidative stress induced by lipopolysaccharide, a bacterial toxin, in mice (Xiao et al. 2021).

##### Toxicity studies of orientin: acute and chronic evaluations

This section reviews acute and chronic toxicity studies of orientin, focusing on metabolic adaptations and safety margins to provide insights into its long-term tolerability and biological impact.

##### Acute toxicity studies

Acute toxicity studies focus on the immediate or short-term effects of orientin upon a single or limited exposure. These studies help determine the initial safety profile by evaluating behavioral changes, physiological responses, and histopathological alterations in major organs (Akharaiyi et al. 2024; Meitasari and Sasongko 2025). Key parameters evaluated include oxidative stress, mitochondrial dysfunction, metabolic alterations, pain relief, and long-term cognitive outcomes. The primary objective of these studies is to detect any toxic effects that may arise, providing a basis for determining the initial threshold of safety (Xiao et al. 2022; Guo et al. 2023).

Experimental findings indicate that orientin exhibits a generally favorable acute toxicity profile, as it does not induce significant systemic toxicity at commonly studied concentrations. Researchers often assess key biomarkers, such as liver and kidney function indicators, to detect any signs of hepatotoxicity or nephrotoxicity. In addition, hematological parameters are evaluated to determine any alterations in blood composition that may suggest immunotoxicity or other systemic disturbances (Aliyu et al. 2020).

Studies indicate that orientin exhibits neuroprotective properties rather than neurotoxic effects, as demonstrated in models of ischemic stroke, where it reduced neuronal injury by mitigating oxidative stress and apoptosis. In addition, comprehensive reviews highlight its antioxidant and anti-inflammatory activities, further supporting its safety profile. These findings suggest that orientin, under acute exposure conditions, does not provoke immediate cytotoxic effects at the cellular or organ level, but rather offers potential neuroprotective benefits (Tian et al. 2018; Jing et al. 2020; Vasudevan Sajini et al. 2024).

##### Chronic toxicity studies

Unlike acute toxicity, chronic toxicity studies aim to assess the long-term safety of orientin upon repeated administration over extended periods. These studies are essential for understanding cumulative toxicity, metabolic adaptations, and potential delayed adverse effects that may not be evident in short-term exposure. Furthermore, a study by Mazibuko-Mbeje et al. (2021) examined how orientin modulates essential genes involved in energy regulation to enhance substrate metabolism. The research indicated that orientin effectively improved metabolic activity, primarily by maintaining substrate utilization, which is marked by enhanced glucose and palmitate uptake. This suggests potential benefits of orientin in managing metabolic disorders, which is relevant to its chronic use.

A recent 2024 review explores the versatile pharmacological activities of orientin, a natural glycoside, highlighting its diverse biological properties. The study examines its antioxidant, anticancer, neuroprotective, and cardioprotective effects, emphasizing their relevance in assessing the compound's long-term safety and potential therapeutic applications (Raza Ishaq 2024). Moreover, genotoxicity assays provide evidence that orientin does not induce DNA damage or chromosomal aberrations, further supporting its long-term safety. A study evaluating a flavonoid-rich extract containing orientin reported no mutagenic effects in the Ames test and no chromosomal aberrations in vitro (Thakurdesai et al. 2023).

##### Mechanisms underlying orientin’s safety profile

The generally low toxicity of orientin can be attributed to its inherent biochemical properties, including its antioxidant activity, anti-inflammatory potential, and efficient metabolic clearance. Flavonoids like orientin exhibit protective effects against oxidative stress and inflammation, which are often implicated in toxicity-related cellular damage. Furthermore, orientin’s ability to modulate key detoxification enzymes suggests that it does not accumulate in tissues over prolonged exposure, reducing the likelihood of chronic toxicity (Khalil et al. 2022b; Qu et al. 2022).

##### Pharmacokinetics, pharmacodynamics, bioavailability, and stability of orientin

The pharmacokinetics and tissue distribution of orientin in plasma and various tissues (heart, liver, spleen, lung, kidney, brain, stomach, and small intestine) of Sprague–Dawley rats were evaluated using the HPLC–UV technique. After being administered intravenously at a dose of 20 mg/kg, orientin was quickly dispersed and eliminated 90 min later. The main organs in rats where orientin was dispersed without accumulating over time were the liver, lung, and kidney. Orientin, on the other hand, failed to cross the blood–brain barrier (Li et al. 2008). In addition, a comparative analysis was conducted to evaluate the pharmacokinetics of orientin *Trollius chinensis* Bunge extract in healthy Sprague–Dawley rats following intravenous administration at a concentration of 10 mg/kg. In rat plasma, orientin's pharmacokinetics differed markedly from those of the extract group. There are no additional components in *T. chinensis* Bunge that could change the elimination of orientin because the initial volume of distribution and clearance were comparable. However, through competitive inhibition, other phytoconstituents in the extract decreased the protein binding profile of orientin, speeding up orientin absorption (Zhao et al. 2018). The pharmacokinetics and tissue distribution of orientin in healthy New Zealand white rabbits were evaluated and confirmed by another investigation. When administered intravenously, intramuscularly, or intraperitoneally, the elimination half-life (t½β) outlasted the distribution half-life (t½α). Orientin is hard to get beyond the blood–brain barrier and is found in high concentrations in the kidney, liver, and lung (Tan et al. 2013). Using fluorescence and absorbance spectroscopy, it was discovered in one research that orientin (concentrations ranging from 3 to 50 μM) exhibited hydrophobic and electrostatic interactions with bovine serum albumin. According to this study, the shape of bovine serum albumin may be affected by the binding of orientin with bovine serum albumin (Toprak and Arık 2014).

Rat liver microsomes (RLMs) and human liver microsomes (HLMs) have been used to assess the bioavailability of orientin. Through uridine 5′-diphospho glucuronosyltransferase (UGT1A1, 1A8, 1A9, and 1A10), orientin was metabolized using HLM- and RLM-mediated glucuronide forms that were typical of Michaelis–Menten kinetics. Biphasic kinetics showed that the RLM caused orientin to be metabolized to its equivalent metabolite. Orientin's permeability in Caco-2 cell monolayers was examined. It was found that the most common method of orientin transport was passive diffusion, which suggests that orientin is well absorbed (Shi et al. 2016).

During manufacturing and storage, a particular study tracked changes in the levels of orientin, iso-orientin, and total polyphenol content in commercially fermented rooibos (FR) and unfermented rooibos (UR) teas. The effects of high (660 mg/L, 0–7 days at 30 °C) and low (0.5 mg/L) concentrations of H_2_O_2_, storage (5, 30, and 40 °C), and pH (pH 3–7) were assessed. These compounds were unaffected by the FR extract manufacturing procedure at any stage (Joubert et al. 2009).

## Conclusion

Plant flavonoids have attracted a lot of interest due to their potential health advantages. Extensive research on orientin's medicinal qualities, including antioxidant, antiaging, anti-inflammatory, vasodilation, cardioprotective, radioprotective, neuroprotective, and antidiabetic actions, may lead to a promising therapeutic effect in the medical area. However, the underlying processes of these therapeutic effects have not been well investigated and remain undetermined. Aside from that, orientin was discovered to have trouble crossing the blood–brain barrier due to its hydrophilicity. Furthermore, at this moment, the majority of orientin biology studies are limited to in vitro and preclinical examinations. There are currently no clinical data available to support the use of orientin in patients. As a result, future research on orientin should focus on the underlying mechanisms, pharmacokinetics, and tissue distribution in the human body in order to build a highly effective medicine with fewer side effects for patients.

## Data Availability

The authors confirm that the data are available within the article.
